# Nrf2-SHP Cascade-Mediated STAT3 Inactivation Contributes to AMPK-Driven Protection Against Endotoxic Inflammation

**DOI:** 10.3389/fimmu.2020.00414

**Published:** 2020-03-10

**Authors:** Hui Gong, Haoran Tai, Ning Huang, Peng Xiao, Chunfen Mo, Xiaobo Wang, Xiaojuan Han, Jiao Zhou, Honghan Chen, Xiaoqiang Tang, Tingting Zhao, Weitong Xu, Chuhui Gong, Gongchang Zhang, Yu Yang, Shuang Wang, Hengyi Xiao

**Affiliations:** ^1^From the Lab for Aging Research, State Key Laboratory of Biotherapy, National Clinical Research Center for Geriatrics, West China Hospital, Sichuan University, Chengdu, China; ^2^Development and Regeneration Key Lab of Sichuan Province, Department of Anatomy and Histology and Embryology, Chengdu Medical College, Chengdu, China; ^3^Department of Immunology, School of Basic Medical Sciences, Chengdu Medical College, Chengdu, China; ^4^Key Laboratory of Birth Defects and Related Diseases of Women and Children of MOE, State Key Laboratory of Biotherapy, West China Second University Hospital, Sichuan University, Chengdu, China

**Keywords:** AMPK, STAT3, Nrf2, SHP, LPS, inflammation

## Abstract

Signal transducer and activator of transcription 3 (STAT3) is implicated in inflammation processing, but the mechanism of its regulation mostly remains limited to Janus kinase (JAK)-mediated phosphorylation. Although AMP-activated protein kinase (AMPK)-mediated STAT3 inactivation has got documented, the molecular signaling cascade connecting STAT3 inactivation and the anti-inflammatory role of AMPK is far from established. In the present study, we addressed the interplay between AMPK and STAT3, and revealed the important role of STAT3 inactivation in the anti-inflammatory function of AMPK in lipopolysaccharide-stressed macrophages and mice. Firstly, we found that pharmacological inhibition of STAT3 can improve the anti-inflammatory effect of AMPK in wild-type mice, and the expression of STAT3 in macrophage of mice is a prerequisite for the anti-inflammatory effect of AMPK. As to the molecular signaling cascade linking AMPK to STAT3, we disclosed that AMPK suppressed STAT3 not only by attenuating JAK signaling but also by activating nuclear factor erythroid-2-related factor-2 (Nrf2), a redox-regulating transcription factor, which consequently increased the expression of small heterodimer protein (SHP), thus repressing the transcriptional activity of STAT3. In summary, this study provided a unique set of evidence showing the relationship between AMPK and STAT3 signaling and explored a new mechanism of AMPK-driven STAT3 inactivation that involves Nrf2-SHP signaling cascade. These findings expand our understanding of the interplay between pro- and anti-inflammatory signaling pathways and are beneficial for the therapeutic development of sepsis treatments.

## Introduction

Bacterial infection often causes acute and severe systemic inflammation, such as endotoxemia and pneumonia (1, 2). Lipopolysaccharide (LPS), also known as endotoxin, is a major pathogenic component of the membrane of gram-negative bacteria. Upon LPS stimulation, innate immune cells, such as monocytes and macrophages, are activated and generate various pro-inflammatory cytokines, including tumor necrosis factor-α (TNF-α), interleukin 1 beta (IL-1β), and inducible nitric oxide synthase (iNOS). These cytokines disturb the balance of cellular metabolism, causing elevated vessel permeability and tissue injury (3, 4). Bacterial infection triggers various inflammatory-associated diseases accompanied by the accumulation of pro-inflammatory cytokines (5). Inhibiting the production of inflammatory cytokines is an important strategy for treating inflammatory diseases.

AMP-activated protein kinase (AMPK), a metabolically sensitive serine/threonine protein kinase, consists of α, β, and γ subunits and is known as the key metabolic regulator of cellular homeostasis; its activity is predominantly regulated by the AMP/ATP ratio in cells (6, 7). AMPK is also a negative mediator of inflammation (8, 9); studies found that AMPKβ1^−/−^ mice exhibited increased infiltration and activation of macrophages in adipose tissue, and the AMPK activator AICAR alleviated inflammation progression in different animal models (10–12). AMPK ameliorates inflammation and inflammatory-associated disease by inhibiting nuclear factor kappa-light-chain-enhancer of activated B cells (NF-κB) target gene expression *in vivo* and *in vitro* (13, 14). Moreover, activation of the anti-oxidative nuclear factor erythroid-2-related factor-2 (Nrf2) pathway by AMPK has been detected in LPS-induced macrophage inflammation (15). It was also found that AMPK has a connection with other inflammatory signaling pathways, including Janus kinase (JAK)/signal transducer and activator of transcription 3 (STAT3) (11, 16).

STAT3 is a member of the STAT superfamily. After phosphorylation of T705 by JAK1/2, STAT3 translocate to the nucleus (17) and promotes the transcription of its target genes, including inflammatory-related cyclooxygenase 2 (*Cox2*) and suppressor of cytokine signaling 3 (*Socs3*) (18, 19). It is generally acknowledged that STAT3 affects the production of pro-inflammatory cytokines by binding and activating the NF-κB subunit RelA/p65 in the nucleus (20, 21) However, as STAT3 deficiency in macrophages and neutrophils causes chronic colitis in mice, it is also assumed that STAT3 is a negative regulator of inflammation (22). Therefore, the exact role of STAT3 in inflammation seems context-dependent (23).

Although previous studies have revealed that STAT3 phosphorylation increases in LPS-stimulated macrophages (24, 25), and AMPK regulates STAT3 deactivation [11, 16], the exact role of STAT3 in the AMPK-STAT3 regulatory pathway is ambiguous in endotoxin-mediated inflammation. In this study, we assessed the following issues: (1) the time-dependent alterations in STAT3 and AMPK activities during LPS-mediated inflammation; (2) the significance of STAT3 in the anti-inflammatory effect of AMPK; (3). the relationship between AMPK and STAT3; and (4) the mechanism by which AMPK suppresses STAT3 activity. Based on *in vitro* and *in vivo* experiments, we confirmed that AMPK negatively regulates STAT3 activity associated with Nrf2-SHP induction and that this AMPK-driven STAT3 suppression contributes substantially to the anti-inflammatory function of AMPK.

## Materials and Methods

### Reagents and Antibodies

Berberine (BBR) was obtained from CDMUST Biotech (Chengdu, China). Lipopolysaccharide (LPS) (*E. coli*) was from Biosharp (Beijing, China). Compound C (CC) was purchased from Calbiochem (Darmstadt, Germany). AG490 was from Sigma (Darmstadt, Germany), S3I-201 and ML385 were purchased from Selleck (CA, USA). Antibodies against phospho-AMPKα1 (T172) (ab133448) and AMPKα1 (ab32047) were from Abcam (MA, USA), those against STAT3 (79D7), phospho-STAT3 (Y705) (D3A7), were from Cell Signaling Technology (BSN, USA), those against phospho-JAK2 (Y1007+Y1008) (CY6570) was from Abways Technology (Shanghai, China), phospho-Nrf2 was from Bioss Antibodies (Beijing, China). FITC-goat anti-rabbit IgG was from Invitrogen (CA, USA). Plasmids transfection reagent was Jet PRIME from PolyPlus (Illkirch, France).

### Cells

RAW264.7 cells (mouse macrophage line) were cultured in RPMI-1640 medium with 10% FBS and maintained in a humidified 5% CO_2_ atmosphere at 37°C.

For peritoneal macrophage preparation, mice were stimulated by a single i.p. injection of 4% thioglycolate solution (1 ml per mouse); peritoneal macrophages were harvested after 4 days by washing peritoneal cavity with PBS (5 ml per mouse), and seeded in RPMI-1640 medium with 10% FBS. Non-adherent cells were removed 2 h after seeding with medium change. Adherent cells were maintained in a humidified 5% CO_2_ atmosphere at 37°C and used for experiments within 7 days (15, 26).

### Mice

ICR mice were provided by DaShuo biotechnology company (Chengdu, China). Control C57 mice (STAT3^flox/flox^) and STAT3 deficient C57 mice (LysMcre/Stat3^flox/flox^) was provided with permission from Dr. Shizuo Akira (Laboratory of Host Defense, WPI Immunology Frontier Research Center, Osaka University). All mice were raised under SPF condition and had free access to food and water. All animal experiments were carried out following protocols approved by the Institutional Animal Care and Use Committee (IACUC) of Sichuan University (approved animal protocol number 2016065A). All protocols adhered to the Guide for the Care and Use of Laboratory Animals.

### RNA Interference

STAT3 siRNA (sense: GGACGACUUUGAUUU CAACTT; antisense: GUUGAAAUCAAAGUCG UCCTT), SHP siRNA (sense: CCAAGACAG UAGCCUUCCUTT; antisense: AGGAAGGCUA CUGUCUUGGTT) were purchased from Sangon Biotech (Shanghai, China). For RNAi experiments, cells were transfected with siRNA using Jet PRIME transfection reagent (PolyPlus). The media were replaced with 1640/10% fetal bovine serum 24 h after transfection, and the cells were used for subsequent experiments.

### Plasmids and DNA Transfection

Dominant negative AMPKα1 plasmid, pDN-AMPKa1 (T172A) was a gift from Dr. Jae Bum Kim; constitutive activated AMPKα1 plasmid, pCA-AMPKα1 (T172D) encodes AA1–312 of AMPKα1 with threonine 172 mutated to aspartic acid was created. Firefly luciferase expressing plasmid with STAT3 promoter (pSTAT3-TA-luc, Cat: D2259) was from Beyotime Biotechnology. pSHP-luc was established by amplifying the mouse SHP promoter (-2K) and inserting it into PGL3-Basic Vector. Endoxin-free plasmids were transfected using Jet PRIME transfection reagent following manufacturer's instruction.

### Promoter Activity Assay

For reporter assays, cells were cultured in 24 well plates, transfected with luciferase reporter plasmid into Raw264.7 cells, with a reference plasmid expressing Renilla luciferase. Following 48 h incubation, luciferase activity was measured using a dual-luciferase reporter system (Promega, Wisconsin, USA).

### Quantitative Real-Time Polymerase Chain Reaction Analysis

Total RNA was extracted from cultured cells or lung tissues of mice using Trizol Reagent (Takara, Shiga, Japan). Reverse transcription for mRNA was carried out using cDNA Synthesis Super Mix (Biotool, cat. B24403). Quantitative PCR was carried out in an ABI cycler using SYBR Green qPCR Master Mix (Biotool, cat. B21203), and the relative amount of cDNA was calculated by the comparative CT method using the 18S ribosomal RNA sequences as control. The primer sequences for PCR amplification are shown in Table 1.

**Table 1:** 18S served as internal normalization control.

**Primer**	**Forward sequence (5′ to 3′)**	**Reverse sequence (5′ to 3′)**
(m)FAS	GTAAGTTCTGTGGCTCCAGAG	GCCCTCCCGTACACTCACTC
(m)CPT-1	ATGGCAGAGGCTCACCAAGC	GATGAACTTCCAGGAGTGC
(m)SOCS3	ATGGTCACCCACAGCAAGTTT	TCCAGTAGAATCCGCTCTCCT
(m)COX2	CCCTGAAGCCGTACACATCA	TGTCACTGTAGAGGGCTTTCAATT
(m)TNF-α	GAAGATGATCTGAGTGTGAGGGT	GCAATACGGACTTGCTCACAGA
(m)IL-1β	AGGATGGGCTCTTCTTCAAAG	GTCTACTGAACTTCGGGGTGAT
(m)iNOS	CAGCACAGGAAATGTTTCAGC	TAGCCAGCGTACCGGATG
(m)SHP	CCATCAGACCGGCCACAAC	AGGTACGCATACTCCTTGGG
(m)NQO-1	GCCTGAGCCCAGATATTGTG	GGAAAGGACCGTTGTCGT
18S	TTGACGGAAGGGCACCACCAG	GCACCACCACCAGGGAATCG

### Immunoblot Analysis

Cells were lysed in RIPA buffer with protease inhibitors cocktail (Biotool, cat. B14002, Houston, TX, USA) for 30 min followed by centrifugation. Fifty microgram of total proteins were loaded on SDS-PAGE gel and separated by electrophoresis, followed by being blotted on PVDF membrane (Millipore, cat. GVWP2932A, Billerica, MA, USA). The target proteins were probed by corresponding primary antibodies with optimized conditions and then incubated with the secondary antibody. Immunological signals were surveyed using Immobile Western Chemi-luminescence HRP substrate kit (Zen Bioscience, cat. 501926) and detected with ECL plus Western Blotting Reagent Pack (Bio-Rad, Hercules, CA, USA). And Image J (Version 1.48v) (Image J software, Bethesda, MD, USA) was used to quantify the protein density of the blots from 3 independent of the experiments. GraphPad Prism (version 5.0) (GraphPad software, San Diego, CA, USA) was used for statistical analysis. The quantitative data were compared using the one-way ANOVA, followed by the Tukey's *post-hoc* test, and *p* < 0.05 was considered significant.

### Pinocytosis Assay

Culture media were removed and washed with PBS for three times, and 0.1% neutral red (in PBS) was added. After 3 h, cells were washed twice with PBS, and then the image was got with an optical microscope or the quantification was obtained with lysis solution (1:1 of 0.1 M acetic acid and 100% ethanol) for 6 h, absorbance was measured at 570 nm with a Spectra (Shell) Reader.

### Animal Experiments

For experiments aiming to ensure the significance of STAT3 inhibition for the effect of AMPK, wild type ICR mice (female, 7–8 weeks) were divided randomly into four groups: (i) LPS, (ii) LPS with BBR, (iii) LPS with S3I-201, and (iiii) LPS with BBR plus S3I-201 (*n* = 6). Mice were injected intraperitoneally with LPS, with saline or BBR, based on the protocol published previously [15]. 5 mg/kg S3I-201 was injected intraperitoneally 2 h later. Six hours after LPS injection, mice were sacrificed under 3% pentobarbital sodium. The blood samples were collected and analyzed by a hematology analyzer; lung tissues were used for RT-PCR assay and hematoxylin eosin staining (HE).

For experiments aiming to reveal the impact of STAT3 deficiency on the effect of BBR, STAT3^flox/flox^ and LysMcre/STAT3^flox/flox^ mice (female, 7–8 weeks) were divided randomly into three groups: (i) saline; (ii) LPS; and (iii) LPS with BBR (*n* = 6). LPS were injected intraperitoneally with saline or BBR. As previously described, 6 h after LPS injection, mice were sacrificed under 3% pentobarbital sodium and the lung tissues were used for quantitative real-time PCR assay and HE staining.

### Histological Analysis

The lung tissues from WT or STAT3 KO mice were collected. Lung tissues were preserved in 4% formaldehyde, bisected at the mid belly and embedded in paraffin perpendicularly with the same polarity. Then, H&E-stained cross sections from lung tissues of each animal were obtained.

### Statistical Analysis

Results represent data from multiple (at least 3) independent experiments. Data are presented as means ± SD; Statistical analysis was performed using the GraphPad Prism (version 5.0) (GraphPad software, San Diego, CA, USA) using Student *t*-test. The differences were significant when *p* < 0.05 and highly significant when *p* < 0.01.

## Results

### The LPS-Induced Inflammatory Response Accompanies an Increase in STAT3 Activity and a Decrease in AMPK Activity

**Figure 1:**
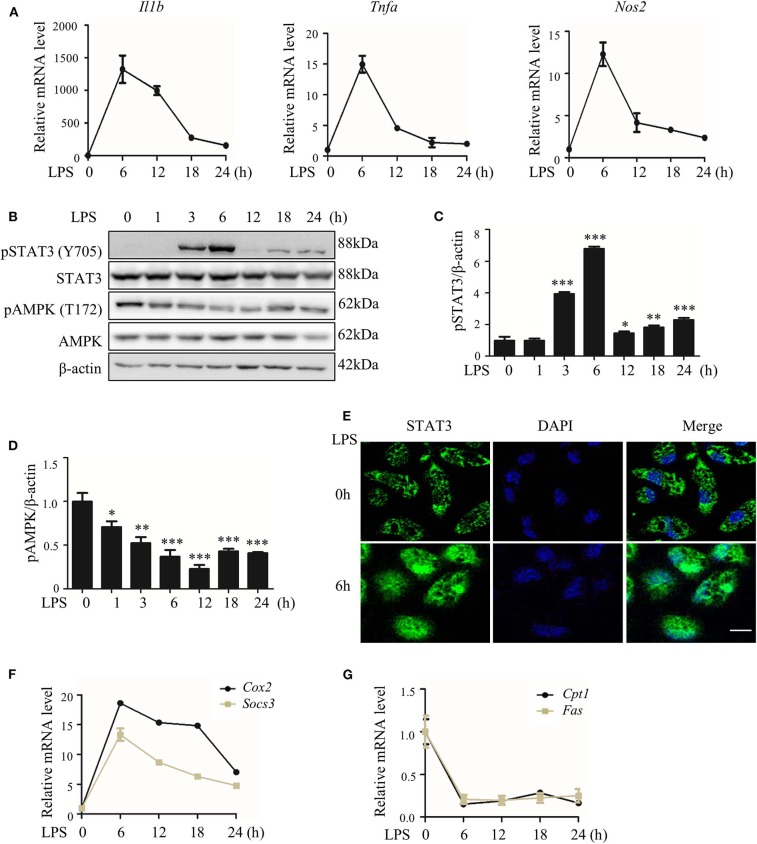
LPS-induced inflammatory response accompanied with altered activities of STAT3 and AMPK. RAW264.7 cells were treated with LPS (100 ng/ml) for indicated hours. **(A)** Relative mRNA levels of *Il1b, Tnfa*, and *Nos2*. **(B)** The level of phosphorylated STAT3 at Y705 site (pSTAT3) and phosphorylated AMPKα at T172 site (pAMPK). **(C,D)** The quantifications of immunoblots from 3 repeats of the experiments. **(E)** Subcellular localization of STAT3 **(F,G)** Relative mRNA levels of *Socs3, Cox2, Cpt1*, and *Fas*. Data are presented as means ± SD from 3 independent experiments. *p < 0.05, **p < 0.01, ***p < 0.001, compared to LPS 0h group. Bar represents 50 μm.

We first collected data showing that LPS treatment quickly induced the expression of pro-inflammatory genes in macrophages (Figure 1A), with synchronized but opposite changes in STAT3 and AMPK activities; STAT3 was activated, but AMPK was inactivated (Figures 1B–G). These changes were represented by the enhanced phosphorylation (Y705) and nuclear translocation of STAT3 protein and increased expression of the STAT3 target genes *Cox2* and *Socs3* (Figures 1B,C,E,F; Figure S5A), together with the reduced phosphorylation (T172) of AMPKα1 protein (Figures 1B,D; Figure S5A) and decreased expression of the AMPK target genes carnitine palmitoyl transferase I *Cpt1*) and fatty acid synthase (*Fas*) (Figure 1G).

Is the relationship between AMPK and STAT3 causal or concurrent? How does this relationship affect inflammation? Our data showed that AMPK activation not only played an anti-inflammatory role in LPS-induced inflammation (Figure S1A), as we previously mentioned (15), but also reduced STAT3 phosphorylation (Figure S1B), similar to other reports (24, 25). In addition, we confirmed the pro-inflammatory impact of STAT3 in LPS-stressed macrophages using genetic and pharmacological approaches (Figure S2). These data together indicate a causal relationship between AMPK and STAT3, suggesting that AMPK-driven STAT3 inactivation may contribute to the anti-inflammatory effect of AMPK.

### STAT3 Inactivation Contributes to the Anti-Inflammatory Effect of AMPK in Macrophages

**Figure 2:**
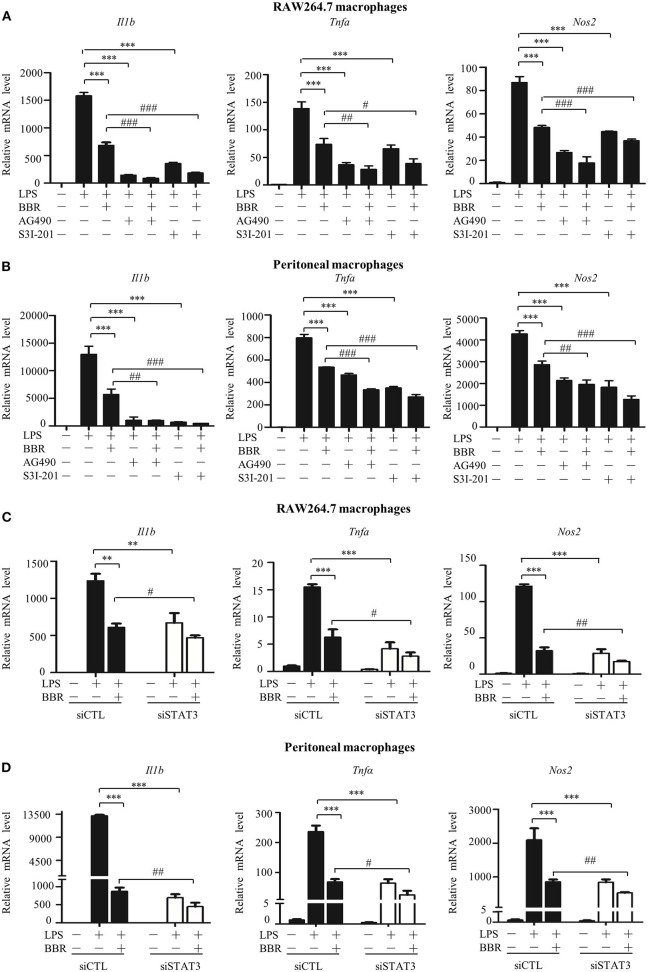
STAT3 inactivation mimics and improves the effect of AMPK in macrophages. **(A–D)** Cells were pre-treated with BBR (10 μM) and AG490 (35 μM) or S3I-201 (50 μM) for 2 h and then with LPS (100 ng/ml) for 6 h. **(A,B)** Relative mRNA levels of the *Il1b, Tnfa*, and *Nos2*. **(C,D)** Cells were transfected with siSTAT3 or siCTL for 36 h, followed by BBR and LPS treatments as described above. Relative mRNA levels of the *Il1b, Tnfa*, and *Nos2*. Data are presented as means ± SD from 3 independent experiments **p < 0.01, ***p < 0.001, compared to LPS alone group; ^#^*p* < 0.05, ^##^*p* < 0.01, ^###^*p* < 0.001, compared to LPS + BBR group.

We assessed the significance and contribution of STAT3 inactivation on the anti-inflammatory effect of AMPK via an *in vitro* approach. Similar to the AMPK activator berberine (BBR), either the JAK2 inhibitor AG490 or the STAT3 DNA binding inhibitor S3I-201 suppressed the expression of pro-inflammatory genes (Figures 2A,B). More intriguingly, both inhibitors improved the anti-inflammatory effect of BBR, showing an enhanced reduction in pro-inflammatory gene expression caused by BBR (Figures 2A,B). Accordingly, STAT3 silencing not only relieved inflammation similar to BBR but also enhanced the anti-inflammatory effect of BBR (Figures 2C,D). Besides, we examined the particle-engulfing activity of macrophages as the defensive function of macrophages. The results showed STAT3 inhibition were more engulfed neutral red particles which was similar with the effect of BBR (Figures S3A,B). These results suggest that blocking LPS-induced STAT3 activation has a similar impact as that of the AMPK activator in inflammation resistance, and furthermore, STAT3 inactivation mimics and improves the effect of the AMPK activator.

### STAT3 Inhibition and AMPK Activation Showed Similar Anti-Inflammatory Effect in Mice

**Figure 3:**
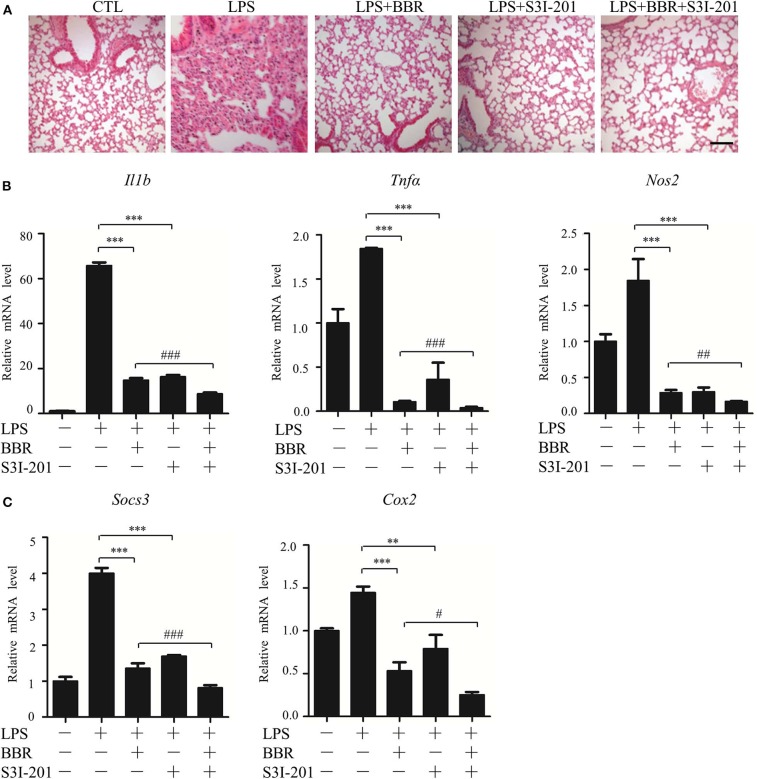
STAT3 inhibition and AMPK activation showed similar anti-inflammatory effect in mice. Mice were intraperitoneally injected with LPS (15 mg/kg) alone or LPS with BBR (10 mg/kg) or S3I-201 (5 mg/kg) and were sacrificed 6 h after LPS injection. **(A)** HE-staining images of lung tissue. Black arrows indicate infiltration of inflammatory cells. **(B)** Relative mRNA levels of inflammatory genes in lung tissue. **(C)** Relative mRNA levels of the STAT3 downstream genes *Socs3* and *Cox2* in lung tissue. Data are presented as means ± SD from 3 independent experiments **p < 0.01, ***p < 0.001, compared to LPS alone group; ^#^*p* < 0.05, ^##^*p* < 0.01, ^###^*p* < 0.001, compared to LPS + BBR group. Bar represents 50 μm.

Next, an *in vivo* approach was used. Similar to BBR, S3I-201 relieved the pulmonary inflammatory injury caused by LPS, such as inflammatory cell infiltration (Figure 3A). Moreover, S3I-201 decreased the expression of pro-inflammatory genes (Figure 3B). Despite these BBR-like actions, S3I-201 improved the inhibitory effect of BBR on the expression of inflammatory genes (Figure 3B). Furthermore, S3I-201 enhanced the effect of BBR reduction of the expression of STAT3-targeted genes (Figure 3C), confirming the inhibitory role of AMPK on STAT3 activity. Together with the data from the *in vitro* experiments, these results demonstrate that STAT3 inactivation and AMPK activation showed similar anti-inflammatory effect in mice with endotoxemia.

### AMPK Activation Fails to Rescue LPS-Induced Inflammation in Myeloid-Specific STAT3-Deficient Mice

**Figure 4:**
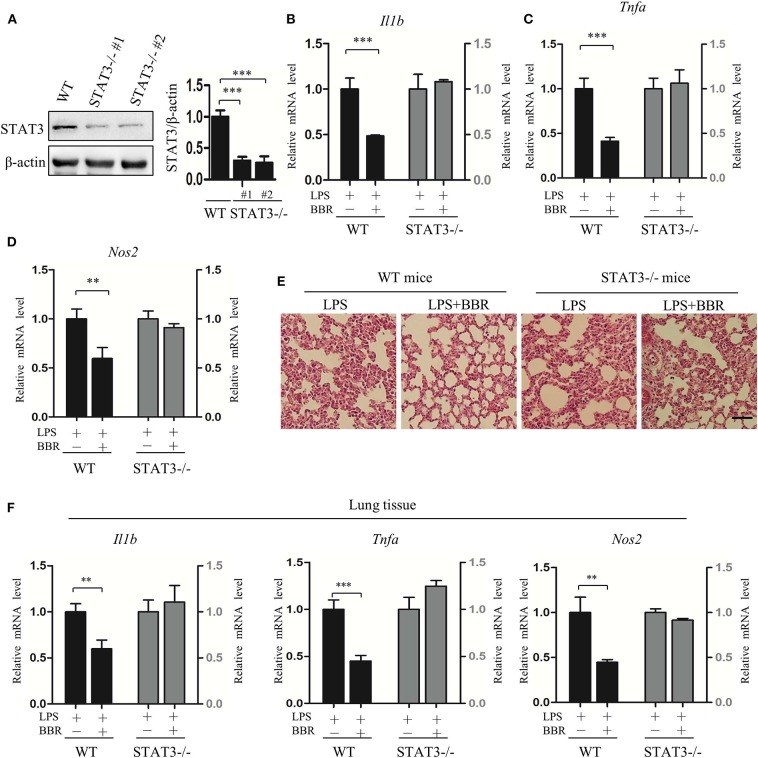
STAT3 deficiency eliminates the anti-inflammatory effect of AMPK in mice. Peritoneal macrophages were obtained from wild-type and STAT3-deficient mice, in which the STAT3 gene was specifically deleted in myeloid cells. Mice were intraperitoneally injected with LPS (15 mg/kg) alone or combined with BBR (10 mg/kg) for 6 h. **(A)** STAT3 protein levels in peritoneal macrophages. WT represents wild-type mice; STAT3^−/−^#1 and STAT3^−/−^#2 are two STAT3-deficient mice. **(B–D)** Relative mRNA levels of inflammatory genes in peritoneal macrophages. **(E)** HE-staining images of lung tissue. **(F)** Relative mRNA levels of inflammatory genes in lung tissue. Data are presented as means ± SD from 3 independent experiments. **p < 0.01, ***p < 0.001 compared to indicated group. Bar represents 50 μm.

To further validate the role of STAT3 inactivation in the anti-inflammatory action of AMPK *in vivo*, another series of experiments was conducted using myeloid-specific STAT3 knockout mice. Firstly, STAT3 deficiency in myeloid cells was conformed (Figure 4A; Figure S6), intriguingly, STAT3 deficiency eliminated the beneficial effect of BBR on inflammation resistance, shown as unchanged pro-inflammatory gene expression in peritoneal macrophages (Figures 4B–D). Further results showing unimproved inflammatory cell infiltration (Figure 4E) and unchanged pro-inflammatory gene expression in lung tissue (Figure 4F). These results suggest that STAT3 expression and response to LPS stimulation is a prerequisite of the anti-inflammatory effect of AMPK in mice.

### AMPK Suppresses JAK-Mediated STAT3 Activation

**Figure 5:**
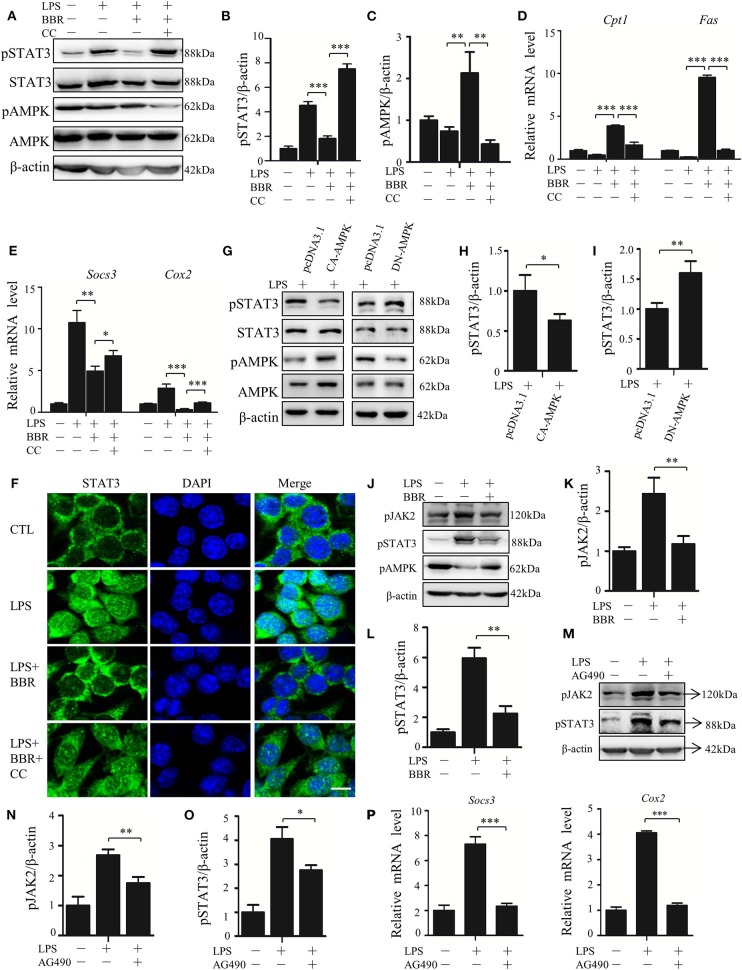
AMPK inhibits STAT3 activity associated with JAK-mediated signal. **(A–D)** RAW264.7 were pre-treated with BBR (10 μM) and Compound C (CC, 10 mM) alone or in combination for 2 h, then treated with LPS (100 ng/ml) for 6 h. **(A)** The level of pAMPK (T172), pSTAT3 (Y705) were shown; **(B,C)** the quantifications of immunoblots from 3 repeats; **(D,E)** relative mRNA levels of *Cpt1, Fas, Socs3*, and *Cox2*; **(F)** subcellular localization of STAT3 in RAW264.7 cells. **(G)** RAW264.7 cells were transfected with constitutive activated AMPKα1 (CA-AMPK) or dominate negative AMPKα1 (DN-AMPK) or the empty plasmid pcDNA3.1 for 36 h, followed by LPS treatment for 6 h, the levels of pAMPK (T172) and pSTAT3 (Y705) were shown; **(H,I)** the quantifications of immunoblots from 3 repeats of the experiments. **(J–P)** RAW264.7 were pre-treated with BBR (10 μM) or AG490 (35 μM) for 2 h, then with LPS (100 ng/ml) for 6 h, **(J)** the protein levels of pAMPK (T172), pJAK2 (Y1007 + Y1008) and pSTAT3 (Y705) were shown; **(K,L)** the quantifications of immunoblots from 3 repeats of the experiments; **(M)** the levels of pJAK2 (Y1007 + Y1008) and pSTAT3 (Y705) were shown; **(N,O)** the quantifications of immunoblots from 3 repeats of the experiments; **(P)** relative mRNA levels of *Socs3* and *Cox2*. Data are presented as means ± SD from 3 independent experiments *p < 0.05, **p < 0.01, ***p < 0.001, compared to indicated group. Bar represents 50 μm.

Next, we addressed the possible mechanism(s) underlying the signaling cascade from AMPK activation to STAT3 inactivation. First, we tested the interference of AMPK activation on JAK/STAT3 signaling, the most well-known signal affecting STAT3 activity. We confirmed that BBR efficiently activated the AMPK pathway (Figures 5A,C,D; Figures S4A, S7A) but decreased the phosphorylation of STAT3 at the Y705 site, a classic target of JAK, and the expression of *Socs3* and *Cox2* genes (Figures 5A,B,E; Figure S4B). BBR also blocked LPS-induced nuclear localization of STAT3 protein (Figure 5F). These AMPK-driven alterations in STAT3 were further confirmed using the AMPK inhibitor Compound C (CC), as it abrogated the effect of BBR on STAT3 inhibition (Figures 5A–F). When a genetic approach was applied, the overexpression of CA-AMPK (constitutively active mutant of AMPKα1) markedly decreased the LPS-stimulated phosphorylation of STAT3 at Y705 (Figures 5G,H; Figure S7B), whereas overexpression of DN-AMPK (inactivated mutant of AMPKα1) enhanced Y705 phosphorylation (Figures 5G,I; Figure S7B). To confirm the upstream function of JAK2 on STAT3 activation, the phosphorylation status of JAK2 and its role were assessed. BBR suppressed the phosphorylation of JAK at Y1007/Y1008 sites (Figures 5J,K; Figure S7C), and AG490, an inhibitor of JAK2, blocked the activity of STAT3 (Figures 5L–P; Figure S7D). In short, these results confirm that AMPK can suppress STAT3 in LPS-stressed macrophages by blocking the JAK-involved mechanism.

### AMPK Activates Nrf2-SHP-Mediated STAT3 Inactivation

**Figure 6:**
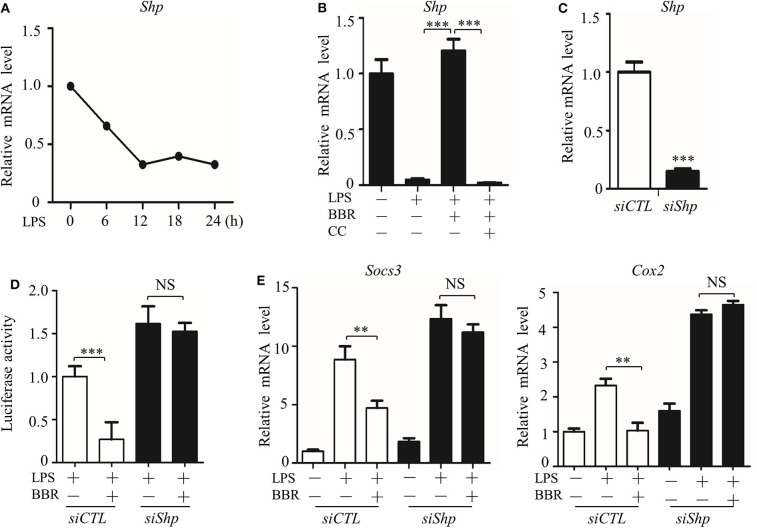
AMPK inhibits STAT3 activity associated with the induction of SHP. **(A)** RAW264.7 cells were treated with LPS (100 ng/ml) for the indicated time. Relative mRNA levels for the *Shp* were shown. **(B)** RAW264.7 cells were treated with LPS for 6 h, alone or combined with BBR (10 μM) or BBR plus CC (10 mM). The relative mRNA level of the *Shp* is shown. **(C)** RAW264.7 cells were transfected with scrambled siRNA (*siCTL*) and *Shp siRNA* (*siShp*) for 48 h, and the relative mRNA level of the *Shp* was shown. **(D,E)** *siCTL* or *siShp* was co-transfected with a Luc-reporter plasmid containing STAT3-binding *cis*-element on its promoter for 48 h, a 6 h treatment of LPS alone or combined with BBR was performed, **(D)** the activity of the *Shp* promoter, and **(E)** the relative mRNA levels of the *Socs3* and *Cox2* are shown. Data are presented as means ± SD from 3 independent experiments. **p < 0.01, ***p < 0.001, compared to indicated group. NS means no significance.

However, we considered whether JAK signaling was the only checkpoint involved in AMPK-mediated STAT3 inactivation. Another molecular mechanism underlying the negative relationship between AMPK and STAT3 was then tested. We evaluated the expression and function of small heterodimer partner (SHP, also referred to as NR0B2) protein, an orphan nuclear receptor, because SHP represses the DNA binding capacity of STAT3, and AMPK upregulates the protein level of SHP (27, 28). We examined whether these phenomena were linked together in our system and how AMPK regulates SHP expression. As shown, parallel with the inactivation of AMPK (Figures 1C,E), *Shp* expression decreased in LPS-stimulated inflammatory macrophages (Figure 6A). In addition, *Shp* expression was upregulated by BBR but downregulated by CC (Figure 6B). To verify the functional association of SHP with the AMPK-STAT3 pathway, we silenced the *Shp* gene (Figure 6C) and found that it markedly abrogated the inhibitory effect of BBR on promoter activity with *cis*-elements for STAT3 binding (Figure 6D) and on the expression of STAT3 target genes (Figure 6E). These results demonstrate that AMPK-induced SHP expression is functionally important for AMPK-induced STAT3 inactivation.

**Figure 7:**
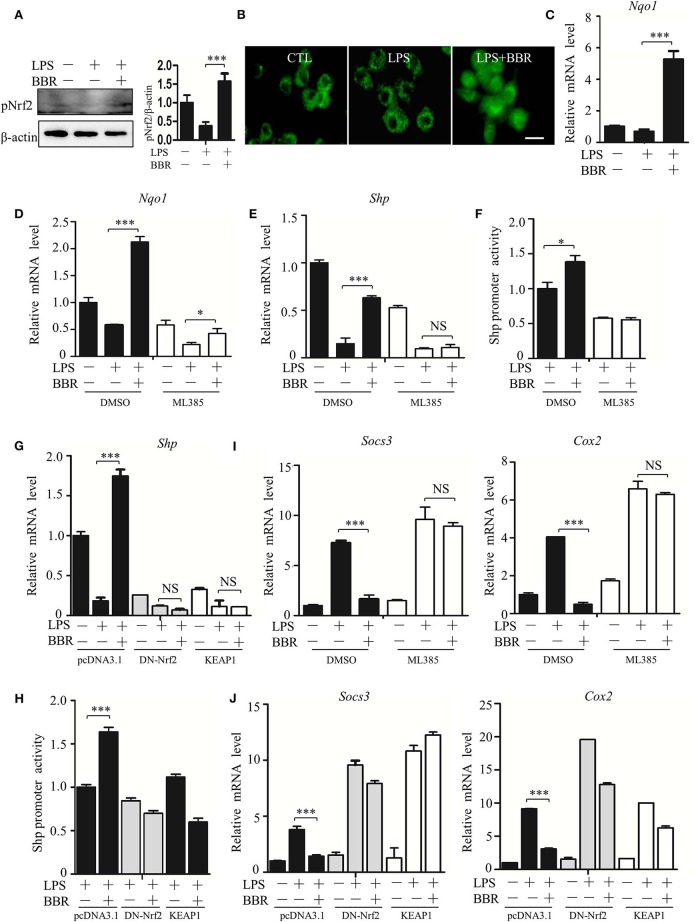
AMPK promotes SHP transcription and STAT3 inactivation through Nrf2. **(A–C)** RAW264.7 cells were treated with LPS and BBR as described in Figure 2. **(A)** The phosphorylation of Nrf2 protein at serine 40 site (S40). **(B)** Immunofluorescent images of Nrf2 in RAW264.7 cells. **(C)** Relative mRNA levels of the Nrf2 target gene *Nqo1*. **(D–F)** RAW264.7 cells were treated with Nrf2 inhibitor ML385 (20 μM), **(D,E)** the relative mRNA levels of *Nqo1* and *Shp*, and **(F)** the activity of the *Shp* promoter. **(G,H)** RAW264.7 cells were transfected with pcDNA3.1 plasmid, S40 mutated Nrf2-expressing construct (DN-Nrf2) and KEAP1 for 36 h, followed by LPS treatment for 6 h. **(G)** The relative mRNA levels of the *Shp*. **(H)** The activity of *the Shp* promoter. **(I,J)** RAW264.7 cells were treated as described in **(D–H)**, and the relative mRNA levels of the STAT3 downstream genes *Socs3* and *Cox2*. Data are presented as means ± SD from 3 independent experiments. NS means no significance. *p < 0.05, ***p < 0.001, compared to indicated group. Bar represents 20 μm.

How does AMPK upregulate the expression of the SHP gene? Based on Nrf2 activation of SHP transcription (29), we inspected the participation of Nrf2 in the signaling cascade from AMPK to STAT3. First, we verified that BBR upregulated the activity of Nrf2 (Figures 7A–C; Figure S8), showing increased phosphorylation, nuclear distribution of NRF2 protein, and elevated expression of its target gene NAD(P)H dehydrogenase [quinone] 1(*Nqo1*). Second, we tested the functional involvement of Nrf2 on SHP expression. The Nrf2 inhibitor ML385 suppressed expression of not only the *Nqo1* gene but also the *Shp* gene (Figures 7D,E). Importantly, this inhibitor subdued BBR-induced expression of the *Nqo1* and *Shp* genes (Figures 7D,E). Moreover, ML385 attenuated the BBR-elevated promoter activity of the *Shp* gene (Figure 7F). Consistent results were obtained from the experiments in which the dysfunctional Nrf2 mutant (DN-Nrf2, S40A) or wild-type kelch-like ech-associated protein 1 (KEAP1) were overexpressed. These results showed that both DN-Nrf2 and KEAP1 overexpression decreased the mRNA level of SHP, repressed the promoter activity of the *Shp* gene and blocked the BBR-induced increase in the mRNA level and promoter activity of the *Shp* gene (Figures 7G,H). Third, we clarified the role of Nrf2 in AMPK-driven STAT3 inactivation, showing that ML385 or KEAP1 and DN-Nrf2 overexpression upregulated STAT3 target genes (Figures 7I,J). More importantly, these factors obstructed BBR-induced STAT3 inactivation (Figures 7I,J). These results indicate that Nrf2 mediates AMPK-driven SHP expression and subsequent STAT3 inactivation.

Altogether, these results reveal a novel signaling cascade that starts from AMPK activation through Nrf2-mediated SHP transcription, resulting in STAT3 inactivation.

## Discussion

LPS-induced inflammation is widely used as a model for elucidating the mechanism underlying acute inflammation because it is a common and pathological occurrence in clinical settings. In addition to the most studied pro-inflammatory molecular cascade, the TLR-NF-κB pathway (30–32), other pro-inflammatory cascades also contribute to the effects of LPS-induced inflammation. Among them, the JAK/STAT3 cascade is of particular importance owing to its close correlation with the bacteria-induced response, and NF-κB-prompted pro-inflammatory cytokine transcription (33, 34). However, the mechanism underlying STAT3 regulation is not fully understood. Interplay between pro- and anti-inflammatory cascades has been recognized as critically important during inflammatory and anti-inflammatory processes. Increasing evidence has revealed the anti-inflammatory role of AMPK (8). However, it is still unclear what the key cascade responsible for this effect is. The antagonistic relationship between STAT3 and AMPK has been hinted at in interleukin 6 (IL-6)-treated murine adipocytes [11], interleukin 4 (IL-4)-treated human monocytes (35), amino acid-treated HepG2 cells (36), and IL-6 treated human hepatocarcinoma [13, 14]. Whether AMPK targets STAT3 during LPS-induced inflammation remains elusive. We obtained lines of evidence verifying the negative regulation of AMPK on STAT3 activity under LPS-induced stress and revealed for the first time that STAT3 inhibition can replace or mask the anti-inflammatory effect of AMPK. Intriguingly, STAT3 deficiency can largely abrogates the effect of AMPK during LPS-induced pulmonary inflammation. Thus, we propose here that AMPK-induced STAT3 inactivation, probably via its indirect effect on NFKB inactivation, plays an important role in the action of AMPK against LPS-mediated inflammation. In other words, the STAT3 cascade is an essential target of AMPK for protection against inflammation.

The most well-known idea regarding this aspect is the AMPK-induced upregulation of phosphorylase1/2 protein (SHP-1/2), which dephosphorylates JAKs and inhibits the capability of JAK to activate STAT3 (37, 38). Bousquet et al. demonstrated that AMPK can activate SHP-2 (*Ptpn11*) by upregulating its gene transcription (37). However, AMPK-induced SHP-1/2 expression was not observed in this study (data not shown). Another mechanism proposed for AMPK-mediated STAT3 inhibition involves AMPK-promoted JAK2 degradation or phosphorylation (39, 40). Our data revealed the reverse correlation between AMPK and the level of Y705 phosphorylation of STAT3 protein, which suggests that JAK2 is involved in AMPK-mediated STAT3 repression.

Interestingly, we identified the Nrf2-SHP pathway as an alternative mechanism underlying AMPK-induced STAT3 inactivation. Our previous s2tudy revealed that AMPK activated Nrf2 signaling, which contributes to the anti-inflammatory effects of metformin and berberine (15). Here, we identified Nrf2 as a mediator of AMPK-mediated STAT3 inactivation. We found that Nrf2 transcriptionally activated the expression of *Shp*, which acts as a transcriptional co-repressor to repress the transcriptional activity of STAT3 (27, 29).

**Figure 8:**
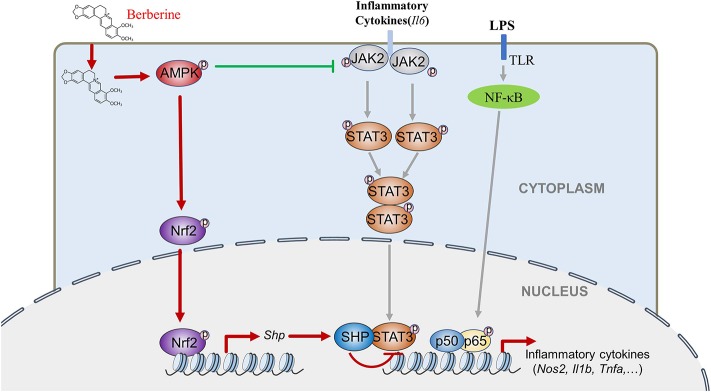
BBR can inhibit LPS-induced inflammation response, which mediated by STAT3 inactivation and Nrf2 activation. While the expression of STAT3 is a prerequisite for the anti-inflammatory effect of AMPK, and AMPK suppresses STAT3 not only by attenuating JAK2 signal, but also by causing the activation of Nrf2 which could increase the expression of small heterodimer protein (SHP), consequently down-regulating the transcriptional activity of STAT3, followed by inflammatory response.

This study indicates for the first time that AMPK induces Nrf2 activation and SHP expression, inactivating STAT3 and contributes to AMPK-driven inflammation restraint. This mechanism is intriguing because it is different from the popular idea that emphasizes AMPK-mediated STAT3 inactivation through dephosphorylating occurring in the cytoplasm. This new one reveals AMPK-mediated STAT3 inactivation via reduced DNA binding capacity occurring in the nucleus. Therefore, AMPK inhibits STAT3 activation via at least two independent mechanisms in LPS-induced inflammation: by decreasing cytokines induced the JAK2 mediated phosphorylation, dimerization and nuclear location of STAT3 that synergistically activates the expression of pro-inflammatory cytokines together with NF-κB, and by promoting the Nrf2/SHP-mediated transcriptional repression of STAT3 (Figure 8). These mechanisms form a redundant system to facilitate AMPK-mediated STAT3 deactivation and inflammation remission. As the initiator of these two pathways, AMPK could be a preferential target for anti-inflammation therapeutics.

In conclusion, this study demonstrated that AMPK can inactivate STAT3 via JAK2-dependent phosphorylation/translocation and Nrf2-SHP-dependent transcriptional repression and that STAT3 is critically involved in AMPK-mediated anti-inflammatory effects *in vitro* and *in vivo*. Therefore, AMPK-mediated STAT3 inactivation plays an important role in the anti-inflammatory effect of AMPK.

## Data Availability Statement

All datasets generated for this study are included in the article/Supplementary Material.

## Ethics Statement

The animal study was reviewed and approved by the Institutional Animal Care and Use Committee (IACUC) of Sichuan University.

## Author Contributions

HG and HT performed most of the experiments, data analyses, and manuscript preparation. HX guided the study planning, experiment processing, and manuscript preparation. NH, PX, CM, XW, and XH conducted experiments. JZ, HC, XT, TZ, and WX performed data analyses. CG, GZ, YY, and SW contributed to the subject discussion. All authors reviewed and approved the final version of the manuscript.

### Conflict of Interest

The authors declare that the research was conducted in the absence of any commercial or financial relationships that could be construed as a potential conflict of interest.

## Supplementary Material

Figure S1 BBR suppresses the expression of inflammation genes and the phosphorylation of STAT3. RAW264.7 cells were pre-treated with berberine (BBR, 10 μM) for 2 h, and then treated with LPS (100 ng/ml) for 6 h. **(A)** Relative mRNA levels of inflammatory genes. **(B)** The levels of pAMPK (T172) and pSTAT3 (Y705) were showed by immunoblots. The data are presented as means ± SD from 3 independent experiments. ^***^*p* < 0.001 compared to LPS alone.

Figure S2 STAT3 prompts LPS-induced inflammation. **(A–C)** RAW264.7 cells were transfected with siSTAT3 or siCTL for 48 h. **(A)** Relative mRNA level of STAT3. **(B)** Total STAT3 protein level. **(C)** Relative mRNA levels of inflammatory genes. **(D)** Cells were transfected with siSTAT3 or siCTL for 36 h, followed by the LPS treatment for 6 h, and relative mRNA levels of inflammatory genes were shown. **(E)** Peritoneal macrophages were transfected with siSTAT3 or siCTL for 36 h, followed by the LPS treatment for 6 h, and relative mRNA levels of indicated genes were detected by qRT-PCR. **(F,G)** Raw264.7 cells were pre-treated with AG490 (35 μM) or S3I-201 (50 μM) for 2 h, then with LPS (100 ng/ml) for 6 h, relative mRNA levels of inflammatory genes were shown. **(H,I)** Peritoneal macrophages cells were pre-treated with AG490 (35 μM) or S3I-201 (50 μM) for 2 h, then with LPS (100 ng/ml) for 6 h, relative mRNA levels of inflammatory genes were shown. The data are presented as means ± SD from 3 independent experiments. ^**^*p* < 0.01, ^***^*p* < 0.001, compared to indicated group.

Figure S3 STAT3 inhibition improve the pinocytosis capacity of macrophages. Cells were pre-treated with BBR (10 μM) and AG490 (35 μM) or S3I-201(50 μM) for 2 h, then treated with LPS (100 ng/ml) for 6 h. Pinocytosis capacity of macrophages was evaluated by neutral red assay. **(A)** Representative images by light microscopy. **(B)** Whole lysates from neutral red stained cells were measured at 570 nm with a Spectra Reader. The data are presented as means ± SD from 3 independent experiments. ^*^*p* < 0.05 compared to LPS alone group; ^#^*p* < 0.05, ^##^*p* < 0.01 compared to LPS + BBR group.

Figure S4 AMPK inhibits STAT3 activity in peritoneal macrophages. Peritoneal macrophages were treated as described in Figure 5, relative mRNA levels of *Cpt1, Fas, Socs3*, and *Cox2* were shown **(A,B)**. The data are presented as means ± SD from 3 independent experiments. ^*^*p* < 0.05, ^**^*p* < 0.01, ^***^*p* < 0.001 compared to indicated group.

Figure S5 Raw western blot records for blots shown in Figure 1.

Figure S6 Raw western blot records for blots shown in Figure 4.

Figure S7 Raw western blot records for blots shown in Figure 5.

Figure S8 Raw western blot records for blots shown in Figure 7.
