# Supplementary figures and images for: Nrf2-SHP Cascade-Mediated STAT3 Inactivation Contributes to AMPK-Driven Protection Against Endotoxic Inflammation
Source: Front Immunol. 2020 Mar 10;11:414. doi: 10.3389/fimmu.2020.00414 (PMC7076194; doi:10.3389/fimmu.2020.00414)

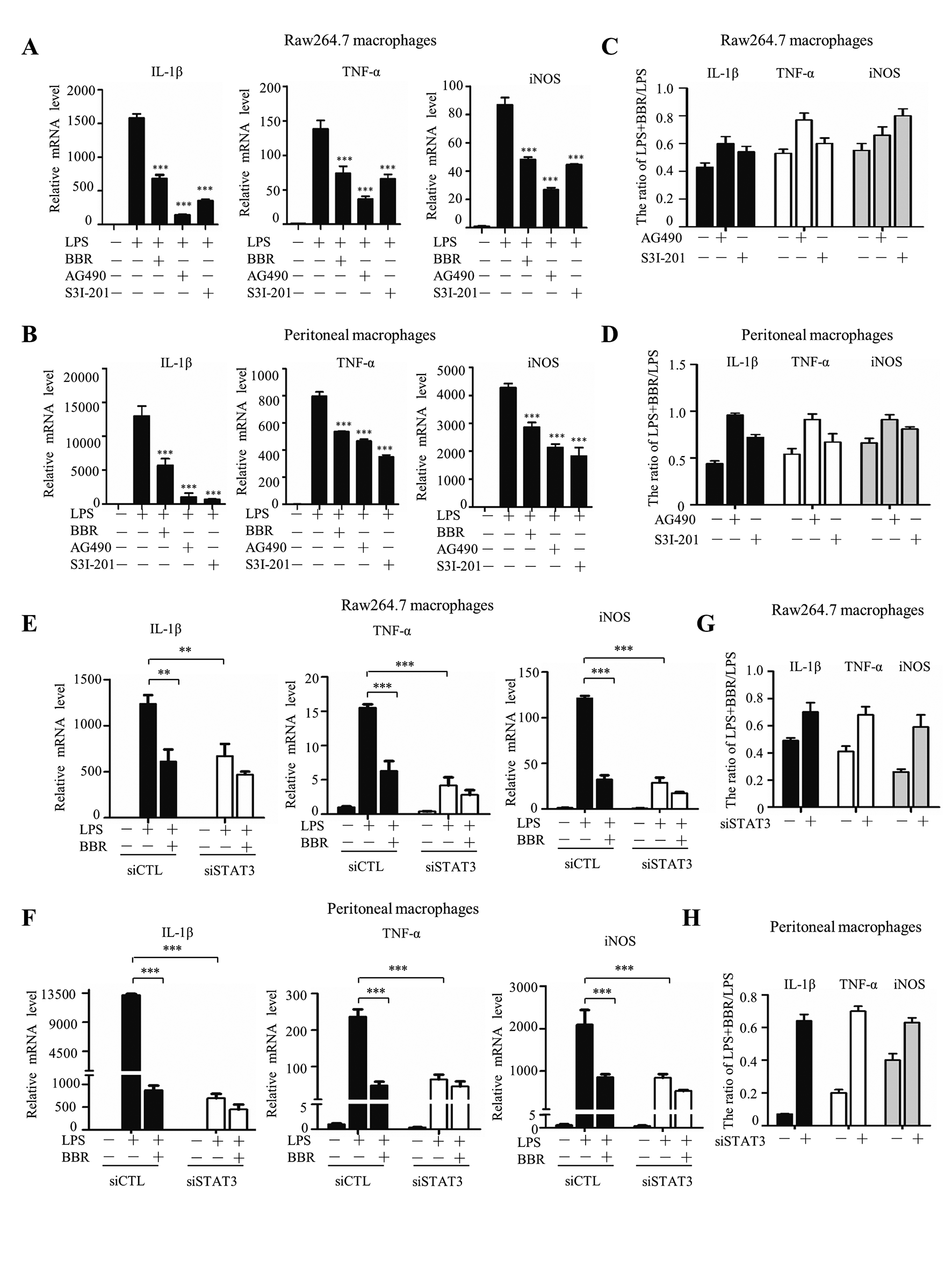

Supplement: Figure S1 — BBR suppresses the expression of inflammation genes and the phosphorylation of STAT3. RAW264.7 cells were pre-treated with berberine (BBR, 10 μM) for 2 h, and then treated with LPS (100 ng/ml) for 6 h. (A) Relative mRNA levels of inflammatory genes. (B) The levels of pAMPK (T172) and pSTAT3 (Y705) were showed by immunoblots. The data are presented as means ± SD from 3 independent experiments. ***p < 0.001 compared to LPS alone. [file Data_Sheet_1.zip › Supplementary figures/Supplementary figure 2.tif]

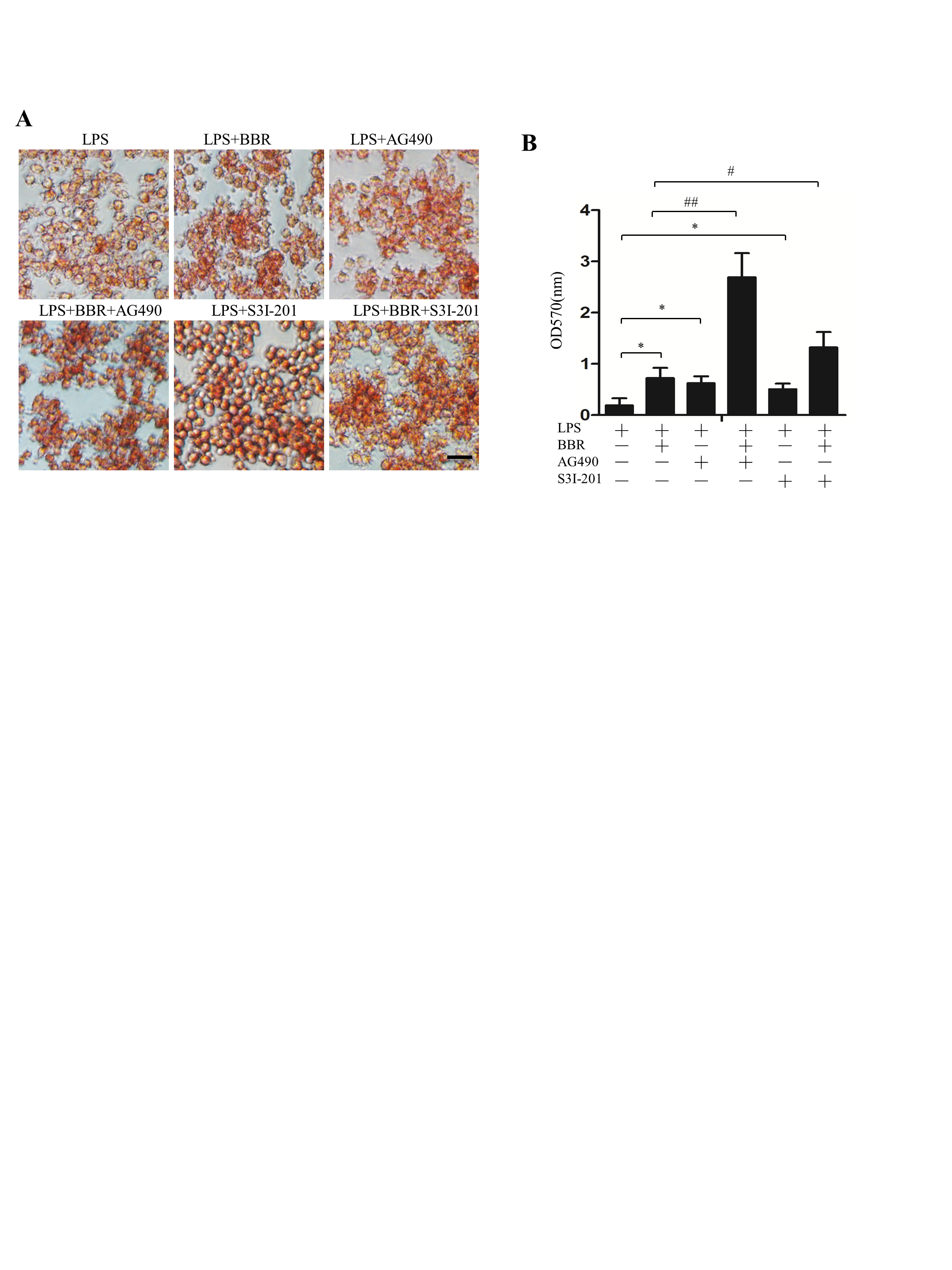

Supplement: Figure S1 — BBR suppresses the expression of inflammation genes and the phosphorylation of STAT3. RAW264.7 cells were pre-treated with berberine (BBR, 10 μM) for 2 h, and then treated with LPS (100 ng/ml) for 6 h. (A) Relative mRNA levels of inflammatory genes. (B) The levels of pAMPK (T172) and pSTAT3 (Y705) were showed by immunoblots. The data are presented as means ± SD from 3 independent experiments. ***p < 0.001 compared to LPS alone. [file Data_Sheet_1.zip › Supplementary figures/Supplementary figure 3.tif]

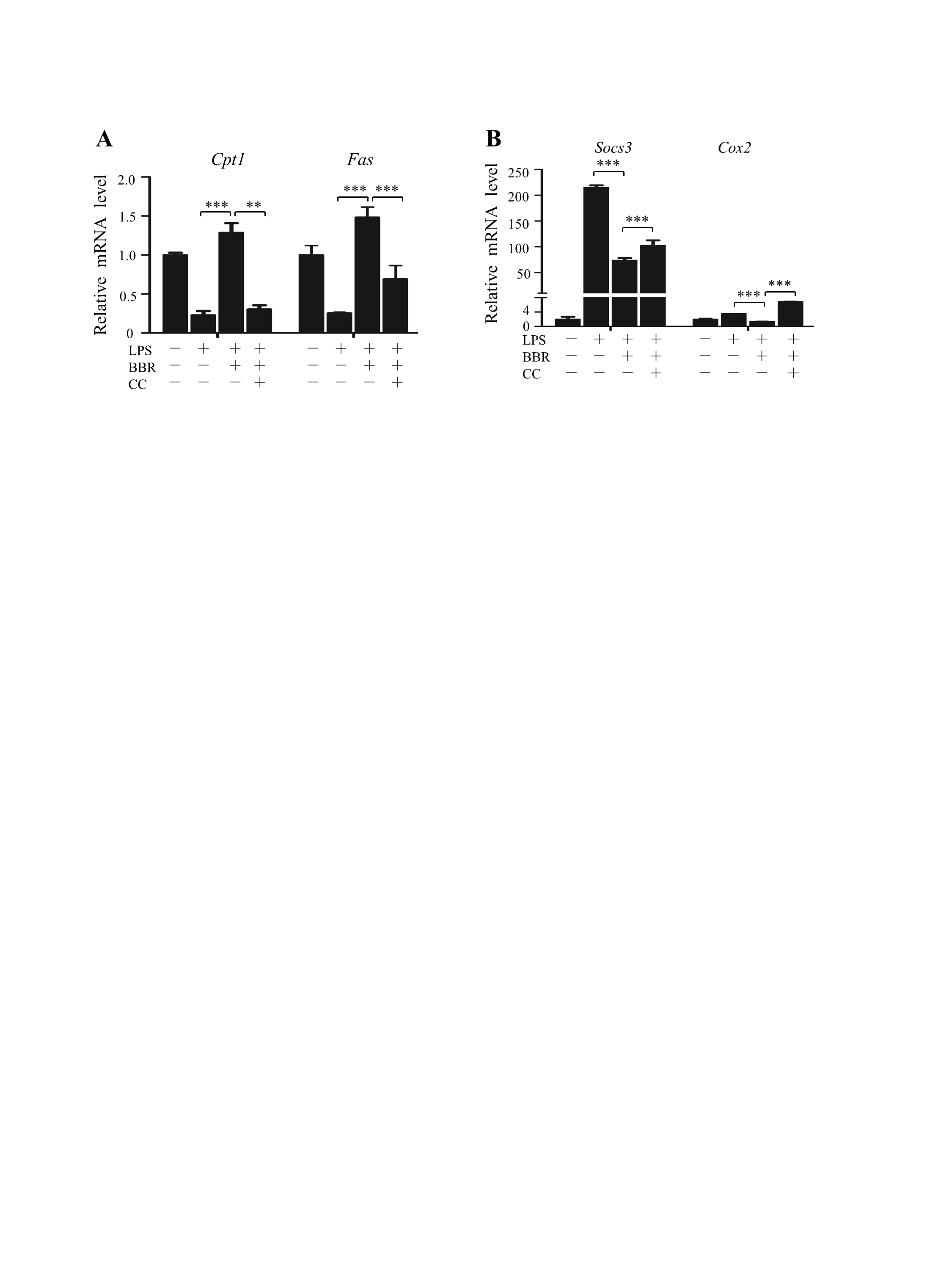

Supplement: Figure S1 — BBR suppresses the expression of inflammation genes and the phosphorylation of STAT3. RAW264.7 cells were pre-treated with berberine (BBR, 10 μM) for 2 h, and then treated with LPS (100 ng/ml) for 6 h. (A) Relative mRNA levels of inflammatory genes. (B) The levels of pAMPK (T172) and pSTAT3 (Y705) were showed by immunoblots. The data are presented as means ± SD from 3 independent experiments. ***p < 0.001 compared to LPS alone. [file Data_Sheet_1.zip › Supplementary figures/Supplementary figure 4.tif]

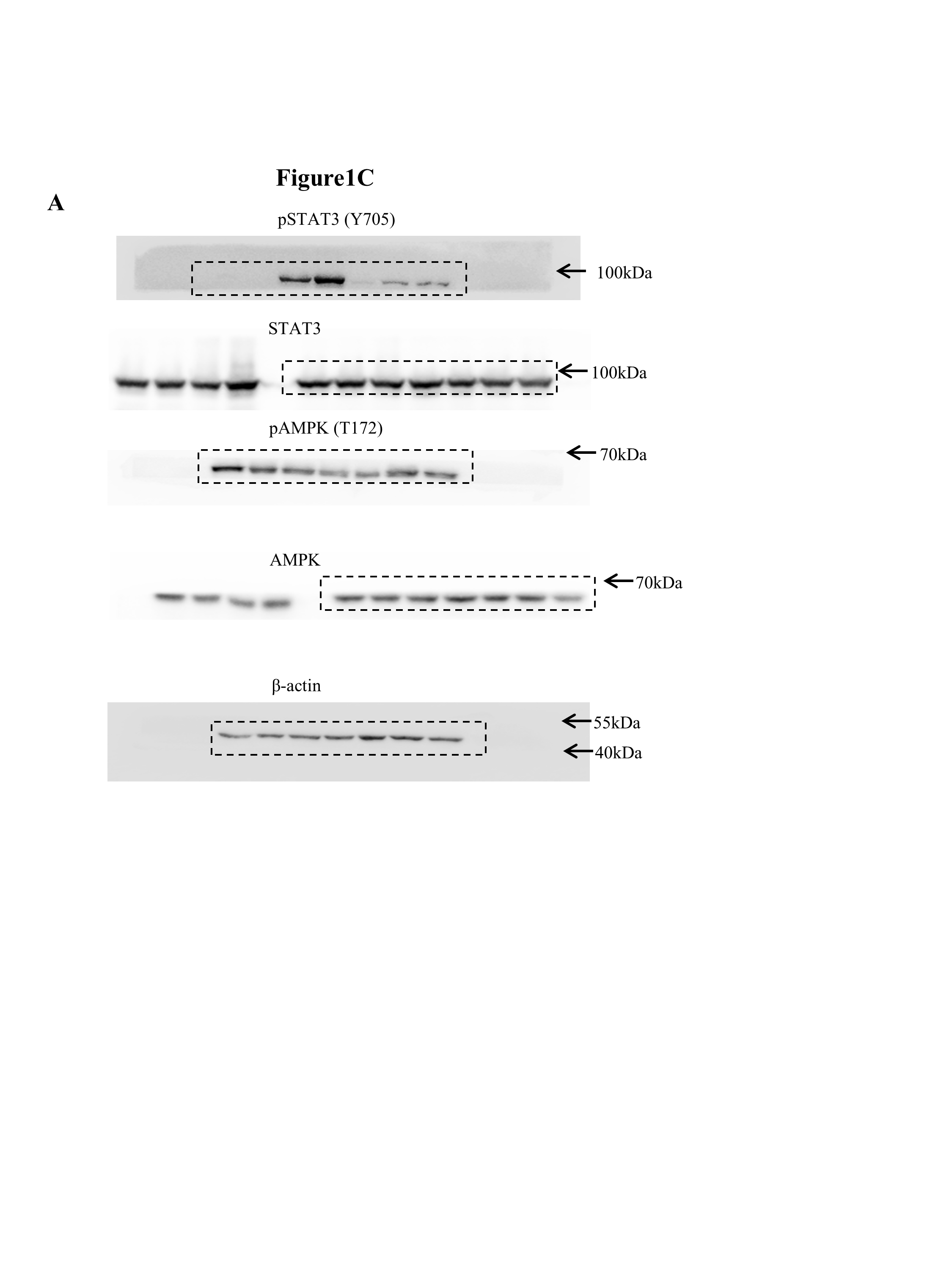

Supplement: Figure S1 — BBR suppresses the expression of inflammation genes and the phosphorylation of STAT3. RAW264.7 cells were pre-treated with berberine (BBR, 10 μM) for 2 h, and then treated with LPS (100 ng/ml) for 6 h. (A) Relative mRNA levels of inflammatory genes. (B) The levels of pAMPK (T172) and pSTAT3 (Y705) were showed by immunoblots. The data are presented as means ± SD from 3 independent experiments. ***p < 0.001 compared to LPS alone. [file Data_Sheet_1.zip › Supplementary figures/Supplementary figure 5.tif]

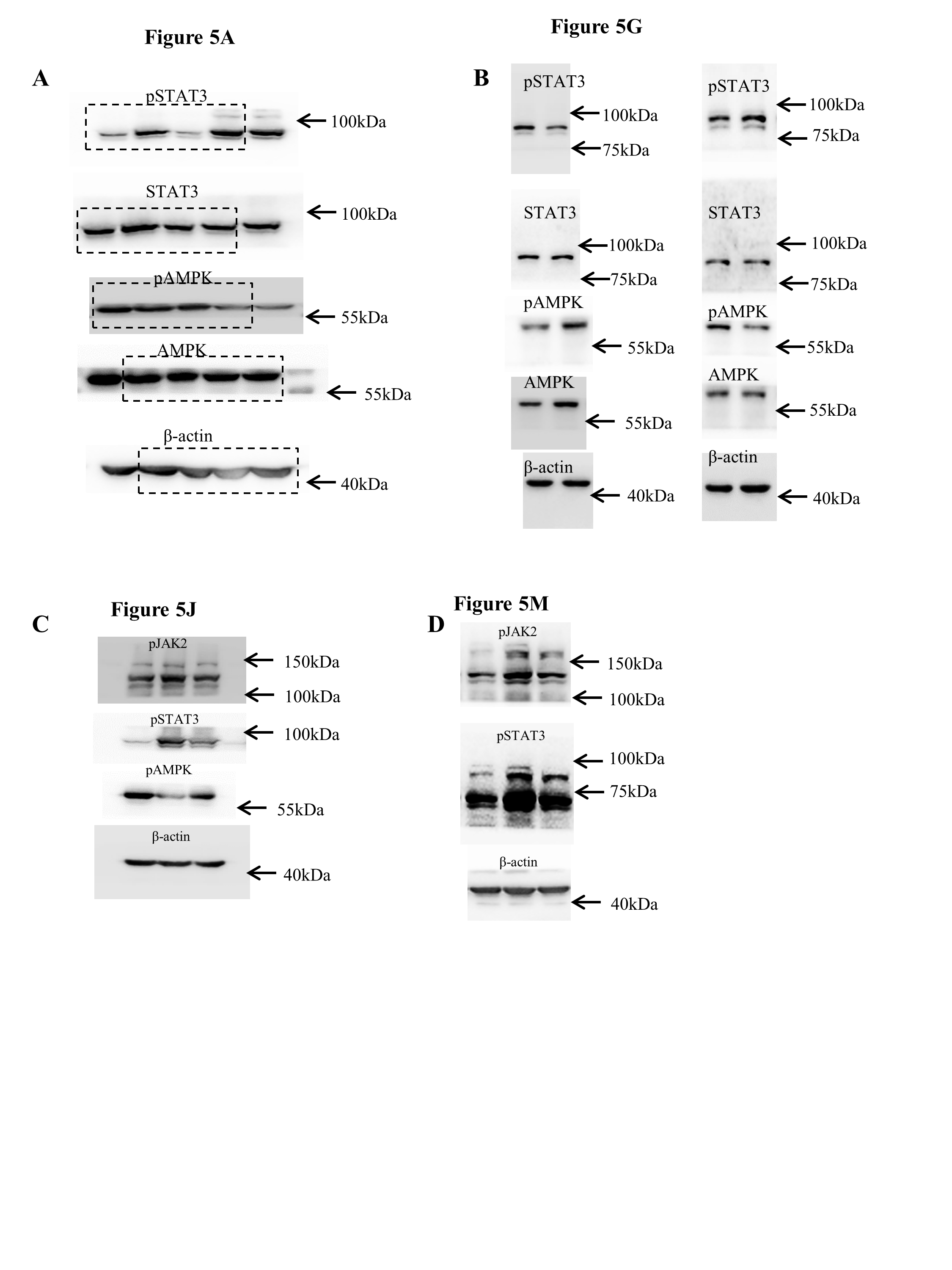

Supplement: Figure S1 — BBR suppresses the expression of inflammation genes and the phosphorylation of STAT3. RAW264.7 cells were pre-treated with berberine (BBR, 10 μM) for 2 h, and then treated with LPS (100 ng/ml) for 6 h. (A) Relative mRNA levels of inflammatory genes. (B) The levels of pAMPK (T172) and pSTAT3 (Y705) were showed by immunoblots. The data are presented as means ± SD from 3 independent experiments. ***p < 0.001 compared to LPS alone. [file Data_Sheet_1.zip › Supplementary figures/Supplementary figure 7.tif]
